# Multiple bilateral oncocytomas of the native kidneys following renal transplantation: report of a rare case and review of the literature

**DOI:** 10.1186/1477-7819-11-119

**Published:** 2013-05-30

**Authors:** Spyridon Vernadakis, George Karaolanis, Demetrios Moris, Vasileios Zavvos, George Liapis, George Zavos

**Affiliations:** 1 Transplantation Unit, LAIKO General Hospital, Athens, Laiko General Hospital, Medical School of Athens, Athens, Greece; 2 Department of Pathology, LAIKO General Hospital, University of Athens, Laiko General Hospital, Medical School of Athens, Athens, Greece

**Keywords:** Renal oncocytoma, Renal masses, Renal transplantation, Renal cell carcinoma

## Abstract

Renal oncocytomas are benign tumors of the kidneys, which are usually diagnosed postoperatively, due to differential diagnostic problems, from a sample of a renal cell carcinoma. The development of a renal oncocytoma in the native kidneys following renal transplantation is a very rare condition and only a few cases have been published in the world literature. In this case report we present a unique case of bilateral multifocal renal oncocytomas of the native kidneys in a female transplant recipient 6 years after renal transplantation. The patient’s postoperative clinical course was uneventful and no local recurrence or distant metastasis has been found so far. The pathology, clinical characteristics, and treatment of renal oncocytomas are also reviewed.

## Background

In 1942, Zippel was the first to describe renal oncocytomas (ROs) [1-4]. ROs are typically uncommon benign, solitary, epithelial tumors with an incidence between 3% to 7% of all primary renal solid tumors [4]. This particular type of tumor has been described in many different organs including the thyroid, salivary glands, parathyroid, pituitary gland, pancreas, adrenal cortex, prostate and kidneys [3,5]. ROs are rarely multiple and/or bilateral. In 95% of cases the tumor is localized unilaterally, while multiple tumors are observed only in 5% of cases [2,4,5]. The common age of observation is the seventh decade of life, with predominance in the male sex (1.6:1). The size of a tumor may vary between 0.6 cm to 14 cm [4]. The oncocytic cell type, which was first described by Hamperl, expresses the electron-microscopic and immunohistochemical characteristics of the intercalated cell type A of the collecting duct and usually grows in an acinar pattern [2,3,6]. Accurate diagnosis and differentiation from the chromophobe type of renal cell carcinoma (RCC) can be challenging and requires microscopic, ultrastructural and immunohistochemical evaluation [2,4]; diagnosis is invaluable in planning treatment.

Development of a RO in the native kidneys following renal transplantation (RT) is very uncommon with only four cases published in the world literature [7-10]. We report herein the first case of a bilateral multifocal RO of the native kidneys in a female transplant recipient 6 years after RT.

## Case presentation

In June 2005, a female patient underwent an ABO-matched deceased-donor RT for end-stage renal disease (ESRD). Her past medical history was significant for chronic renal disease for 11 years, possibly as a consequence of long-standing poorly controlled hypertension and hypercholesterolemia with resulting atherosclerotic disease. She required hemodialysis over the preceding 8 years. During the pre-transplant evaluation, ultrasound examination revealed a few simple renal cysts throughout the reduced-size kidneys and no solid masses could be detected. The gallbladder was free of calculi. The initial immunosuppressive regimen consisted of cyclosporine A (CsA), mycophenolate mofetil (MMF) and prednisone according to our protocol at the time. The donor was a 69-year-old female with no history of tumors and good renal function.

**Figure 1:**
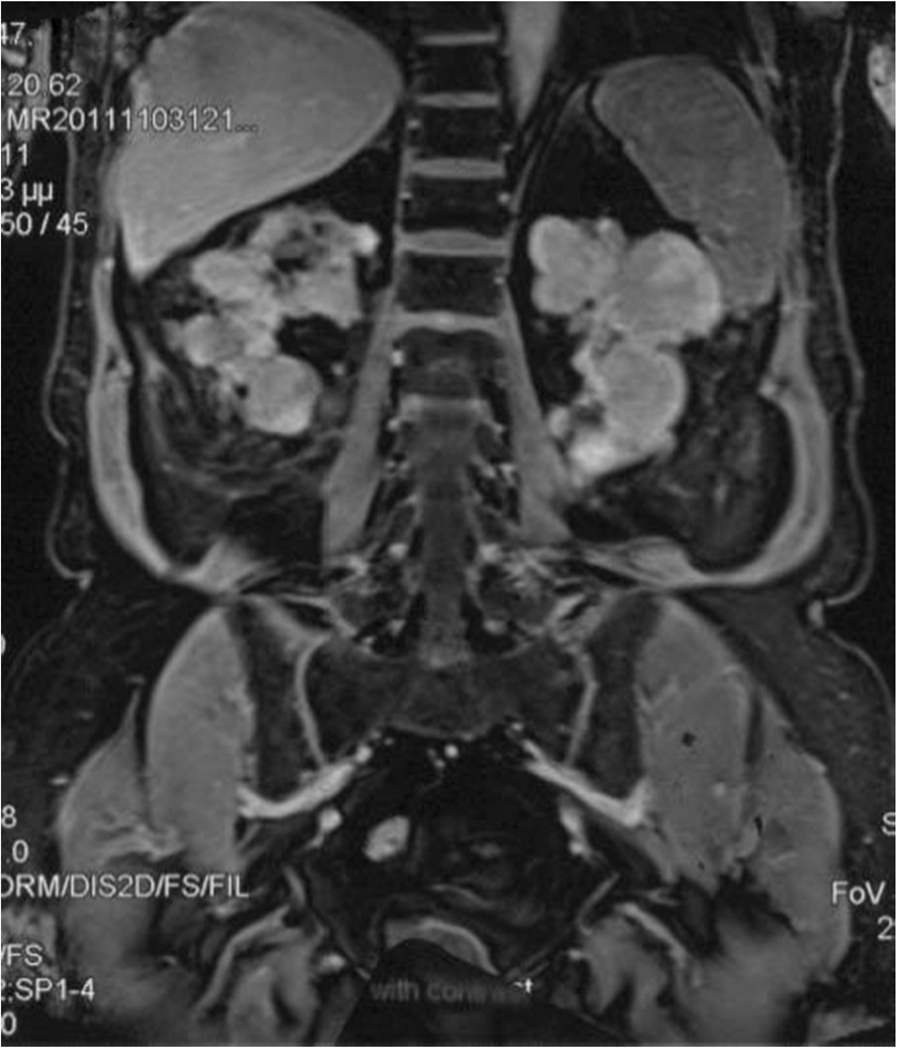
**T1-weighted coronal images.** MRI after contrast enhancement shows multiple bilateral masses with intermediate signal.

Six years after RT, the patient presented with epigastric pain and abdominal discomfort over the right upper quadrant. Laboratory investigations, including kidney and liver biochemical profiles, were within normal range. Ultrasonography demonstrated microlithiasis of the gallbladder, without any signs of acute cholecystitis or dilatation of the biliary tree. There were multiple solid, low-echogenic and inhomogeneous masses incidentally confirmed in both native kidneys. Normal renal transplant perfusion was demonstrated by means of color-coded duplex sonography of the renal graft. Abdominal magnetic resonance imaging (MRI) was performed, which revealed multiple bilateral solid renal masses without any obvious lymphadenopathy or vascular involvement (Figure 1).

A percutaneous biopsy was non-diagnostic due to the lack of vital tissue. Due to the multiplicity of the lesions and the concern that it was a renal cell carcinoma, in November 2011 the patient, now 64 years old, underwent a transabdominal bilateral nephrectomy and simultaneous cholecystectomy.

**Figure 2:**
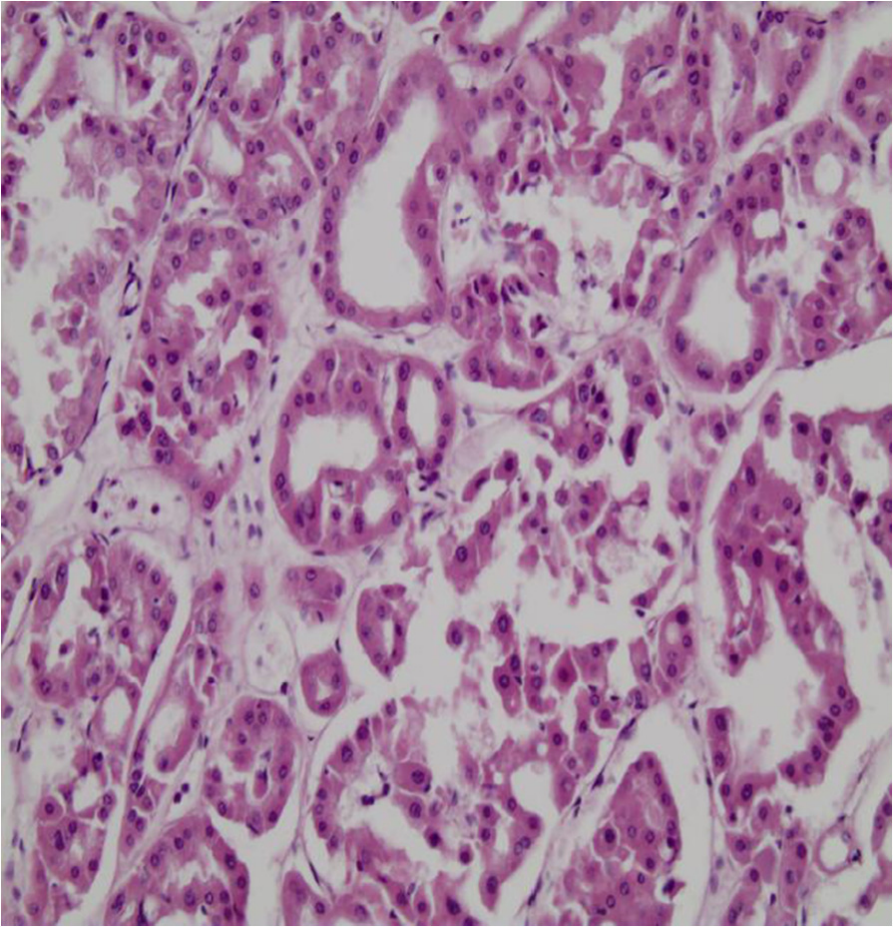
Tubular arrangement of the neoplastic oncocytic cells in an edematous stroma.

Histological assessment revealed multiple bilateral renal oncocytomas (oncocytomatosis) ranging in size from 1 cm to 7.5 cm, replacing most of the renal parenchyma and pelvis of both kidneys. Additionally small cystic changes were identified. Surgical margins and the perirenal fat were free of tumor. The neoplastic cells had an eosinophilic, granular cytoplasm and monomorphic round vesicular nuclei (Figure 2). Immunohistochemical staining for vimentin and CD10 was negative, while CK7 was locally expressed within the tumor masses.

The postoperative course was uneventful and the patient was discharged home on the ninth postoperative day. Currently the patient is tumor free and with normal renal function. No further abnormalities were found at a follow-up CT scan 12 months after the nephrectomy of her native kidneys.

## Discussion

ROs are classified as benign renal neoplasms in the 2004 World Health Organization classification of renal tumors [11]. The tumor originates in the intercalated cells of the distal collecting tubules. Several genetic abnormalities have been associated with this type of tumor, such as deletions in chromosome 1 and loss of chromosome Y or translocations involving chromosomal area 11q13 [4]. A family predisposition to bilateral renal oncocytomas has been observed [2]. Bilateral multicentric ROs are rare and diffuse ROs are even rarer. In 1982, Warfel and Eble described renal oncocytomatosis after identifying more than 200 foci of ROs in one patient [11,12]. The term ‘oncocytomatosis’ should be strictly applied to cases in which oncocytomas infiltrate the whole kidneys, as in our case [13], and correct diagnosis is particularly problematic. Cases of metastatic tumors have been documented [6,14], but subsequent reports have identified subtypes of renal tumors with similar histological morphology but malignant potential. These tumors have been classified as either eosinophilic variants of RCC or chromophobe carcinomas [6]. ROs usually appear to be unifocal, but a multifocal and bilateral appearance and concomitant RCC have been reported [2,5,15-20].

On the other hand, RT remains the best treatment option for ESRD patients, regarding their survival and quality of life. Solid-organ transplant recipients are considered to be at a significant risk of *de novo* malignancies related to immunosuppressive therapy. The reported incidence rate of *de novo* malignancy after RT ranges from 3% to 26%, depending on the length of follow-up, pre-transplant renal disease diagnosis, age distribution of the patients, nature of the applied immunosuppression regimen and the type of malignancy; this incidence is quite high compared to that of the general population. The most common forms of malignancy within renal transplant recipients are skin malignancies and non-Hodgkin lymphomas [21]. However, carcinomas of the native kidneys represent less than 5% of all malignancies found in renal transplant recipients [22]; for this group of people the risk of developing a RCC in the native kidneys, especially in those with acquired cystic kidney disease (ACKD), is 15 times higher than in the normal population [23].

The differential diagnosis for bilateral, solid renal masses includes RCCs, lymphomas, angiomyolipomas, oncocytomas and metastatic disease. For our patient, a bilateral RCC was the leading diagnostic concern given the multiple bilateral solid renal masses especially in the context of the RT. The radiographic presentation of RCC is variable, depending on multiple factors including size, location, extent of necrosis and hemorrhage [2]. A RO was not specifically considered as a leading diagnostic differential during the patient workup, due to the rarity of a bilateral and multifocal growth pattern for a RO.

The diagnostic sequence for a RO is the same as for other renal tumors, but no imaging technique offers absolute certainty of RO diagnosis. CT scan and arteriography findings such as hypodense enhancement after intravenous contrast medium injection, a spoke-wheel vascular formation pattern, a central scar area and the absence of a rounded edge, may suggest the presence of a RO [2,15]. However, these features can be present in a RCC. Moreover, no differentiation using MRI is possible [24]. On the other hand, radiology-guided bioptic procedures can be useful for diagnosis, but they contain the risk of seeding tumor cells. Especially for multifocal, bilateral solid tumors, as in our case, it is not possible to establish a safe diagnosis.

Microscopically, ROs are characterized by round or polygonal cells that are eosinophilic epithelial cells called oncocytes with a mitochondria-rich cytoplasm arranged in trabecular or tubular patterns [2,4,10]. Histological differential diagnosis from a chromophobe RCC is very important and can be challenging. The presence of cytokeratin 7 would hint at a RCC. Expression of cytokeratin 20 would hint at a urothelial neoplasm [15].

The differential diagnosis of native kidney solid tumors in renal transplant recipients is difficult and challenging. It is typically an incidental finding during ultrasound examination or a CT scan for other clinical conditions. Furthermore, only four cases of renal transplant recipients with oncocytomas in the native kidneys have been reported in the literature. Rostaing *et al*., and Cofán *et al*. [7,8] each describe one RT recipient, who developed a solitary oncocytoma in a native kidney up to 9 years post RT. Moreover, Turney and Harnden [9] reported another solitary oncocytoma of a native kidney in a short-term transplant recipient observed 1 year post RT. Riehl *et al*. [10] discuss a young renal-graft recipient who developed a solitary RO in a native kidney with ACKD, 4 years after RT. In this case report, we present a unique case of renal oncocytomatosis of the native kidneys in a renal transplant recipient with normal graft function, 6 years following RT.

## Conclusions

Until a pre-surgical diagnosis is available, surgery is the first-line treatment. The classical approach to a solid renal mass has been in general a radical nephrectomy in cases with adrenal gland involvement, due to the high probability of the lesion being a RCC. In solitary tumors less than 4 cm in diameter a nephron-sparing procedure (open or laparoscopic partial nephrectomy) is recommended [25]. In cases with a bilateral multifocal RO, radical nephrectomy could be advised.

## Consent

Written informed consent was obtained from the patient for publication of this case report and any accompanying images. A copy of the written consent is available for review by the editor-in-chief of this journal.

## Abbreviations

ACKD: Acquired cystic kidney disease; CsA: Cyclosporine A; ESRD: End-stage renal disease; MMF: Mycophenolate mofetil; MRI: Magnetic resonance imaging; RCC: Renal cell carcinoma; RO: Renal oncocytoma; RT: Renal transplantation.

## Competing interests

The authors declare that they have no competing interests.

## Authors’ contributions

SV, GZ and DM participated in the design of this study and drafted the manuscript. GZ performed the operation. GK and VZ carried out the study and collected important background information. GK was involved in the postoperative follow-up. GL conceived this study and participated in the design. All authors read and approved the final manuscript.
